# Sulfhydryl Modification Induces Calcium Entry through IP_3_-Sensitive Store-Operated Pathway in Activation-Dependent Human Neutrophils

**DOI:** 10.1371/journal.pone.0025262

**Published:** 2011-10-03

**Authors:** Leiting Pan, Xian Wu, Dan Zhao, Nason Ma’ani Hessari, Imshik Lee, Xinzheng Zhang, Jingjun Xu

**Affiliations:** The MOE Key Laboratory of Weak-Light Nonlinear Photonics, TEDA Applied Physics School and School of Physics, Nankai University, Tianjin, People’s Republic of China; Cornell University, United States of America

## Abstract

As the first line of host defense, neutrophils are stimulated by pro-inflammatory cytokines from resting state, facilitating the execution of immunomodulatory functions in activation state. Sulfhydryl modification has a regulatory role in a wide variety of physiological functions through mediation of signaling transductions in various cell types. Recent research suggested that two kinds of sulfhydryl modification, S-nitrosylation by exogenous nitric oxide (NO) and alkylation by N-ethylmaleimide (NEM), could induce calcium entry through a non-store-operated pathway in resting rat neutrophils and DDT_1_MF-2 cells, while in active human neutrophils a different process has been observed by us. In the present work, data showed that NEM induced a sharp rising of cytosolic calcium concentration ([Ca^2+^]_c_) without external calcium, followed by a second [Ca^2+^]_c_ increase with readdition of external calcium in phorbol 12-myristate 13-acetate (PMA)-activated human neutrophils. Meanwhile, addition of external calcium did not cause [Ca^2+^]_c_ change of Ca^2+^-free PMA-activated neutrophils before application of NEM. These data indicated that NEM could induce believable store-operated calcium entry (SOCE) in PMA-activated neutrophils. Besides, we found that sodium nitroprusside (SNP), a donor of exogenous NO, resulted in believable SOCE in PMA-activated human neutrophils via S-nitrosylation modification. In contrast, NEM and SNP have no effect on [Ca^2+^]_c_ of resting neutrophils which were performed in suspension. Furthermore, 2-Aminoethoxydiphenyl borate, a reliable blocker of SOCE and an inhibitor of inositol 1,4,5-trisphosphate (IP_3_) receptor, evidently abolished SNP and NEM-induced calcium entry at 75 µM, while preventing calcium release in a concentration-dependent manner. Considered together, these results demonstrated that NEM and SNP induced calcium entry through an IP_3_-sensitive store-operated pathway of human neutrophils via sulfhydryl modification in a PMA-induced activation-dependent manner.

## Introduction

As the first line of host defense against invasion of pathogenic microbes, neutrophils play a crucial role in a variety of inflammatory responses [1], [2]. In generally, researchers have known that there are two main states for neutrophils: resting and activation. The activation of the human neutrophils leads to a spectrum of responses, including aggregation, stimulation of the respiratory burst and degradation of microbes. During the transition of neutrophils from resting to activation state, the increase of cytosolic calcium concentration ([Ca^2+^]_c_), due to the calcium release and calcium entry, has been considered as the major intracellular modification factor [3], [4]. As a second messenger, [Ca^2+^]_c_ executes profound effects across a wide range of physiological functions and signaling transductions in neutrophil immunity responses, including the initiation of cytoskeletal changes, degranulation, adhesion, apoptosis, and oxidative burst [5]. In particular, store-operated calcium entry (SOCE), a critical mechanism of [Ca^2+^]_c_ regulation in non-excitable cells [6]-[8], plays a crucial role in modulating the immune responses of neutrophils [9]–[11].

Sulfhydryl groups, a key component of proteins, can be modified by different agents through oxidation, reduction, or alkylation. These modifications play a significant role in various signaling transductions though affecting the activity and structure of different kinds of enzymes, receptors, and ion channel proteins [12], [13]. For instance, N-ethylmaleimide (NEM), a irreversible sulfhydryl-alkylating agent, has long been used for the chemical modification of sulfhydryl groups of cysteine residues in various cells types [14]–[16]. More recently, studies have indicated that nitric oxide (NO) conveys a large part of ubiquitous influence via S-nitrosylation of sulfhydryl groups for providing redox-based physiological regulation [17]. S-Nitrosylation of proteins, a modification of cysteine residues by NO [18], first demonstrated by Stamler [19], performs an important regulatory role in cell apoptosis [20], ion channel activation [21], mitochondrial caspases modulation.

For resting rat neutrophils, Wang *et al* demonstrated that alkylating agent NEM and NO donor 5-amino-3-(3,4-dichlorophenyl)-1,2,3,4-oxatriazolium (GEA3162) just stimulated calcium entry through a non-store-operated pathway via direct sulfhydryl modification [22]–[24]. In addition, Gill *et al* reported that NEM and membrane-permeant NO donors could activate non-store-operated calcium entry through sulfhydryl modification in DDT_1_MF-2 cells [25]–[27]. However, in phorbol 12-myristate 13-acetate (PMA)-activated human neutrophils, we have previously shown that NO donor sodium nitroprusside (SNP) induced calcium release from inositol 1,4,5-trisphosphate (IP_3_) receptor-sensitive stores via S-nitrosylation of sulfhydryl groups [28]. These reports indicated that sulfhydryl modification had different effects on [Ca^2+^]_c_ of activated neutrophils as compared to resting neutrophils. The present study investigated the calcium entry mechanism induced by the alkylation agent NEM and the exogenous NO donor SNP in PMA-activated human neutrophils. We asked whether the effects of NEM and SNP on [Ca^2+^]_c_ were due to the activation of neutrophils. Data showed that NEM or SNP-triggered [Ca^2+^]_c_ increase was due to a IP_3_ receptor-sensitive calcium release and SOCE via sulfhydryl modification in PMA-induced activation-dependent human neutrophils.

## Results

### TG or FMLP induces SOCE in PMA-activated neutrophils

**Figure 1:**
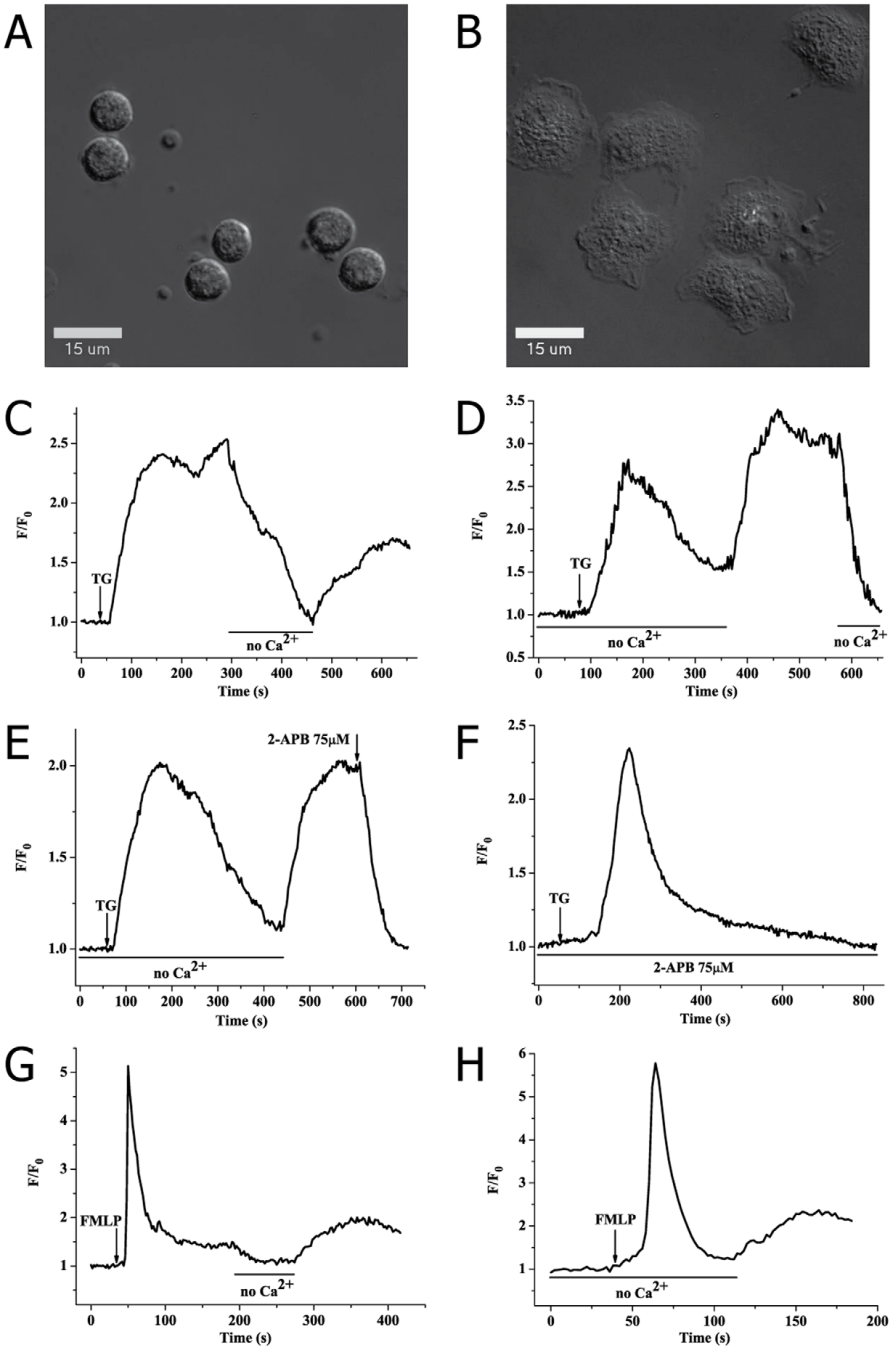
SOCE still occurs but is blocked by 2-APB in PMA-activated human neutrophils. (A) Spherical resting neutrophils visualised by DIC. (B) When PMA was added (2 µM for 5 min at 37°C), neutrophils were activated and spread on glass surface thereby flattening their morphology as observed by DIC. (C) After calcium store depletion by a calcium pump blocker TG (2 µM), external Ca^2+^ (1**mM) was transiently removed 4 min later; then, further addition of 1 mM external Ca^2+^ resulted in obvious calcium entry, revealing the putative response for SOCE. (D) 2 µM TG was added in the absence of Ca^2+^, followed by readdition of 1 mM external Ca^2+^. (E) After calcium stores depletion by 2 µM TG, PMA-activated neutrophils were treated with 75 µM 2-APB. (F) 2 µM TG was added together with 75 µM 2-APB. (G, H) Calcium stores were triggered with G protein-coupled agonist FMLP (2 µM) in the presence/absence of calcium followed by removal/readdition of 1 mM external Ca^2+^.

PMA, a potent protein kinase C (PKC) activator, can activate NADPH oxidase by stimulating PKC and subsequently induce respiratory burst in neutrophils [29], [30]. In our experiments, resting human neutrophils were activated by incubation with 2 µM PMA for 5 min at 37°C, which caused adhesion to the glass surface resulting in observable flattening of the cells with differential interference contrast (DIC) microscopy (Fig. 1A and B). To determine whether the mechanism of SOCE was in use after PMA activation, we measured the [Ca^2+^]_c_ responses to thapsigargin (TG) [31], [32] and N-formyl-methionyl-leucyl-phenylalanine (FMLP) [33], [34], two powerful activators of SOCE in neutrophils. Figure 1 showed the effects of TG and FMLP on [Ca^2+^]_c_ of PMA-pretreated neutrophils. Addition of 2 µM TG induced a sharp rising of [Ca^2+^]_c_ due to calcium store release in Ca^2+^-containing medium, followed by a second extended rising of [Ca^2+^]_c_ (Fig. 1C). The latter peak represented extracellular calcium entry and was abolished when the medium was replaced with nominally Ca^2+^-free HBSS as shown in Figure 1C and D. Moreover, readdition of 1 mM external calcium evidently resulted in a repeat of calcium entry (Fig. 1C and D), indicating the familiar response for SOCE. As illustrated in Figure 1G and H, FMLP (2 µM) showed the same effects on [Ca^2+^]_c_ in the presence or absence of external calcium in PMA-activated neutrophils. In addition, when 75 µM 2-Aminoethoxydiphenyl borate (2-APB) was added during the entry phase, it rapidly and obviously abolished calcium entry (Fig. 1E). When 75 µM 2-APB was added together with TG, it had little effect on the calcium release but completely inhibited the calcium entry component of [Ca^2+^]_c_ in Ca^2+^-containing buffer (Fig. 1F). It is well known that 2-APB is a potent membrane-permeant antagonist of IP_3_ receptor [35]–[38] and a reliable blocker of SOCE [9], [38], [39]. Taken together, these results clearly indicated that the SOCE mechanism still occurred in PMA-activated neutrophils.

### NEM or SNP induces SOCE via sulfhydryl modification in PMA-activated neutrophils

**Figure 2:**
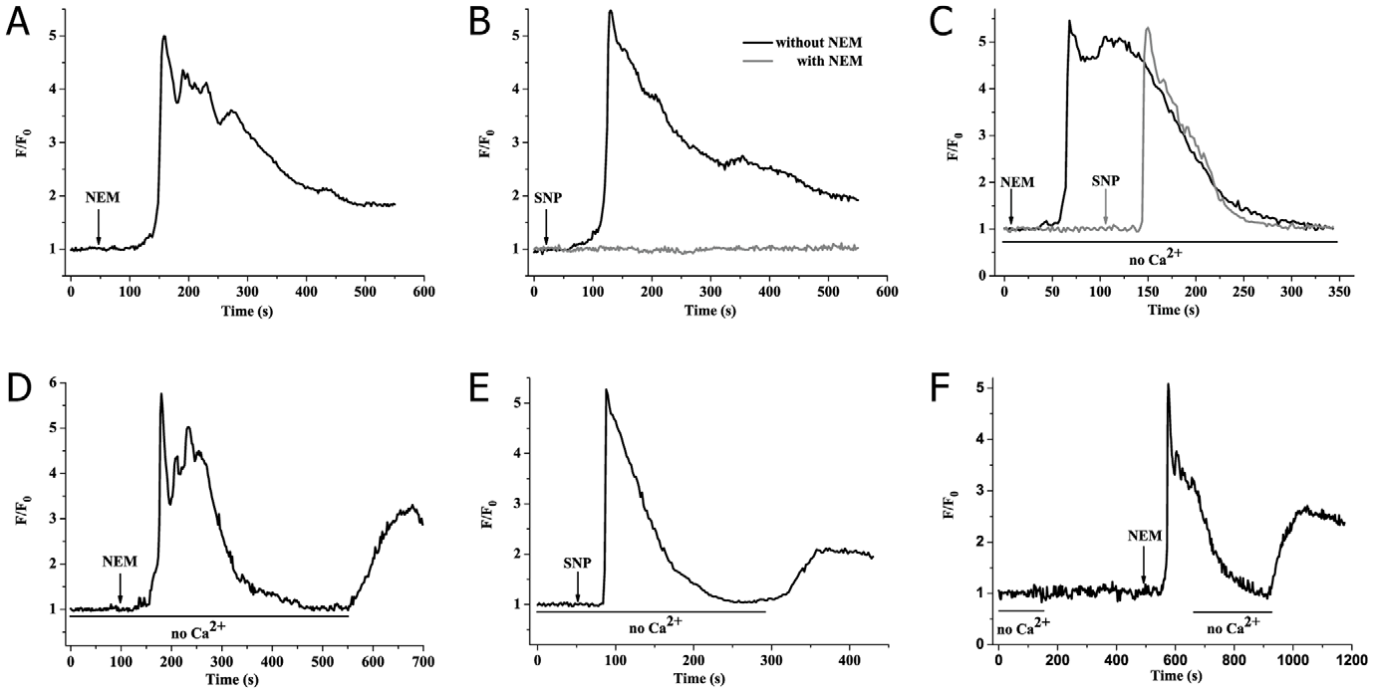
NEM or SNP results in SOCE via sulfhydryl modification in PMA-activated neutrophils. (A) Representative tracing of [Ca^2+^]_c_ elevation induced by 100 µM NEM in the presence of external Ca^2+^. (B) Representative tracings of 500 µM NEM inhibitory effect on [Ca^2+^]_c_ change caused by SNP (500 µM) within Ca^2+^-containing medium. (C) Typical tracings of [Ca^2+^]_c_ responses for PMA-activated neutrophils to 100 µM NEM or 500 µM SNP stimulation in Ca^2+^-free buffer. (D, E), [Ca^2+^]_c_ of PMA-pretreated neutrophils was triggered with NEM (100 µM) within Ca^2+^-free HBSS followed by readdition of 1 mM external Ca^2+^. (F) 1 mM external calcium was added to Ca^2+^-free PMA-activated neutrophils before application of stimulation, followed by addition of 100 µM NEM.

As shown in Figure 2A and B, NEM (100 µM) or SNP (500 µM) induced a rapid [Ca^2+^]_c_ increase followed by a second extended rising of on [Ca^2+^]_c_ in PMA-preincubated neutrophils within Ca^2+^-containing HBSS. When cells were pretreated with NEM (500 µM), the effect of SNP on [Ca^2+^]_c_ was significantly abolished (gray line in Fig. 2B). Our previous work [28] also demonstrated that application of 4H-8-bromo-1,2,4-oxadiazolo(3,4-d) benz (b-1,4) oxazin-1-one (NS2028), a specific inhibitor of soluble guanylate cyclase, had no effect on SNP-induced the rising of [Ca^2+^]_c_ in PMA-activated neutrophils. These results showed that two kinds of sulfhydryl modification, alkylation by NEM and S-nitrosylation by SNP, could cause obvious [Ca^2+^]_c_ increase in PMA-activated neutrophils.

Moreover, in the absence of external Ca^2+^, the increase of [Ca^2+^]_c_ was still observed with the latter peak being completely blocked, indicating that calcium stores release and calcium entry were co-existing (Fig. 2C). To further explore the relationship between calcium stores release and calcium entry, [Ca^2+^]_c_ were triggered with NEM (100 µM) or SNP (500 µM) in the absence of Ca^2+^ followed by readdition of external Ca^2+^ in PMA-activated neutrophils. It was found that subsequent addition of external Ca^2+^ resulted in a further calcium entry event after stimulation by NEM or SNP, demonstrating calcium entry should be store-depletion-dependent (Fig. 2D and E). Besides, addition of 1 mM external calcium had no effect on [Ca^2+^]_c_ of Ca^2+^-free PMA-activated neutrophils before application of stimulation (Fig. 2F). In summary, NEM and SNP could induce believable SOCE via sulfhydryl modification in PMA-activated neutrophils.

### The effect of NEM or SNP on [Ca^2+^]_c_ of resting human neutrophils

**Figure 3:**
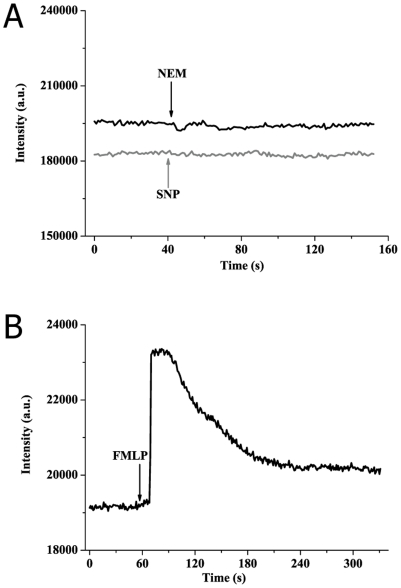
NEM and SNP have no effect on [Ca^2+^]_c_ of resting human neutrophils. (A) Representative tracings of [Ca^2+^]_c_ responses to 100 µM NEM (black line) and 500 µM SNP (gray line). (B) Typical tracing of [Ca^2+^]_c_ change induced by 1 µM FMLP. For all experiments, resting human neutrophils were performed in suspension (1×10^6^/mL) within Ca^2+^-containing buffer.

To further determine whether SOCE induced by NEM and SNP was due to the activation of neutrophils, experiments were performed with resting human neutrophils in suspension (1×10^6^/mL) using spectrofluorometer. It was found that NEM (100 µM) or SNP (500 µM) did not caused [Ca^2+^]_c_ increase with Ca^2+^-containing buffer in resting neutrophils as shown in Figure 3A. However, FMLP (2 µM), a classic activator of calcium mobilization in neutrophils [33], [34], induced evident rising of [Ca^2+^]_c_ in resting neutrophils served as a positive control (Fig. 3B). Considered together, these results suggested that NEM and SNP-induced SOCE via sulfhydryl modification was dependent on activation of neutrophils.

**Figure 4:**
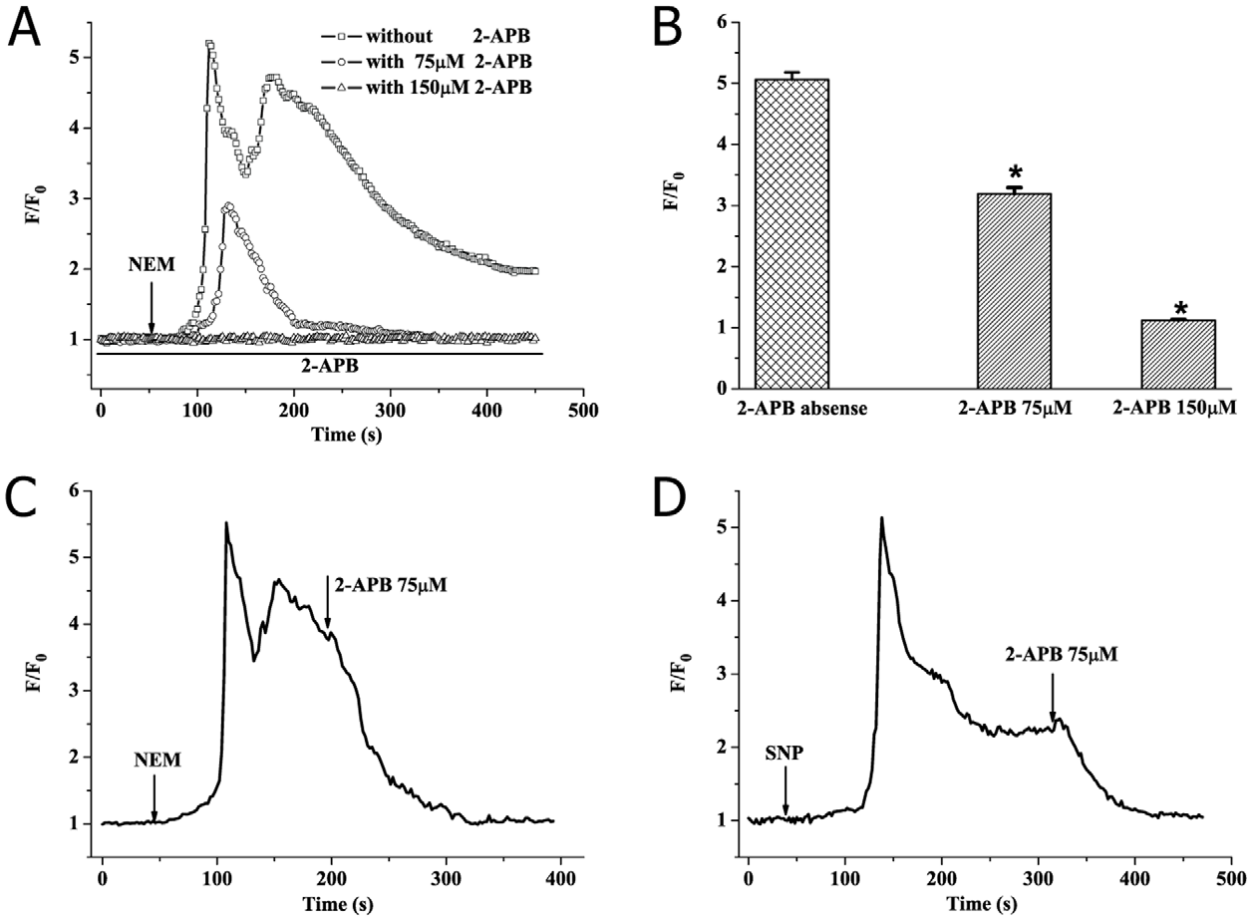
2-APB completely blocks SOCE in response to sulfhydryl modification in PMA-activated human neutrophils. All experiments were performed with cells in Ca^2+^-containing medium. (A) Typical tracings of [Ca^2+^]_c_ changes of PMA-activated neutrophils induced by 100 µM NEM in the presence of 2-APB at 0 µM (-□- curve), 75 µM (-○- curve), 150 µM (-△- curve), respectively. (B) Statistic data from three independent experiments (means±SEM, n = 30 cells). ^*^ *P*<0.01, compared with 2-APB absence. (C, D) [Ca^2+^]_c_ increase was induced by NEM (100 µM) or SNP (500 µM) in PMA-activated neutrophils, followed by addition of 75 µM 2-APB at t = 197 or t = 314 s, resulting in return to baseline [Ca^2+^]_c_.

### The effect of 2-APB on NEM or SNP-induced SOCE in PMA-activated neutrophils

To further study these calcium stores release and calcium entry responsible for sulfhydryl modification-induced SOCE in PMA-activated neutrophils, we measured [Ca^2+^]_c_ responses to the potent IP_3_ receptor [35]–[38] and SOCE [9], [38], [39] blocker 2-APB in Ca^2+^-containing medium. It was found that addition of NEM (100 µM) caused no increase in [Ca^2+^]_c_ elevation when cells were preincubated with 150 µM 2-APB as shown in Figure 4A (-△- curve) and B (maximal value of F/F_0_: 5.062±0.121 for 2-PAB absence vs. 1.121±0.013 for 150 µM 2-APB, n = 30 cells, *P*<0.01). This result indicated that calcium release came from IP_3_ receptor-sensitive stores. Meanwhile, application of 75 µM 2-APB prior to NEM stimulation completely blocked the calcium entry (-○- curve in Fig. 4A) but partially inhibited calcium release (maximal value of F/F_0_: 5.062±0.121 for 2-APB absence vs. 3.191±0.103 for 75 µM 2-APB, n = 30 cells, *P*<0.01; Fig. 4B). 2-APB also had the same effect on SNP-induced [Ca^2+^]_c_ elevation in PMA-activated neutrophils (data not shown). In addition, addition of 75 µM 2-APB to cells already responding to NEM or SNP resulted in rapid decrease in [Ca^2+^]_c_ back to basal levels (Fig. 4C and D). Together, these results demonstrated that 2-APB could totally inhibit calcium entry at 75 µM while blocking calcium release in a concentration-dependent manner, indicating that NEM or SNP induced calcium entry via depletion of IP_3_ receptor-sensitive stores.

### The effect of FMLP or U73122 on sulfhydryl modification-induced SOCE in PMA-activated neutrophils

**Figure 5:**
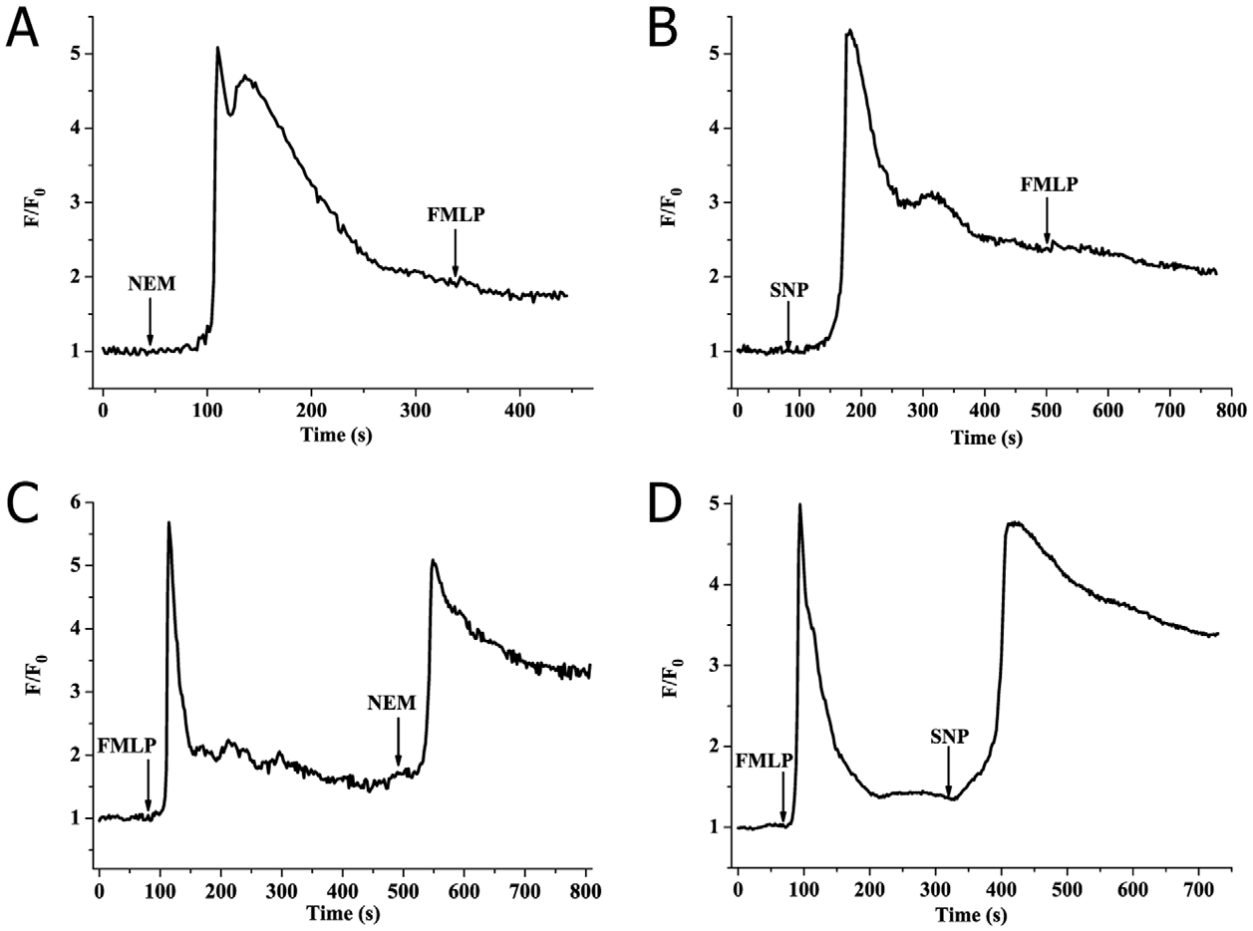
No further calcium elevation occurs with further addition of FMLP when PMA-activated neutrophils were pretreated NEM or SNP, while addition of NEM or SNP after FMLP stimulation still results in rising of [Ca^2+^]_c_. (A, B) Representative tracings of [Ca^2+^]_c_ elevation induced by 2 µM FMLP when cells were pretreated NEM (100 µM) or SNP (500 µM) in PMA-activated neutrophils. (C, D) Typical tracings of [Ca^2+^]_c_ elevation caused by 100 µM NEM or 500 µM SNP after stimulation of FMLP (2 µM).

The classic G protein-coupled receptors (GPCRs)-G protein-Phospholipase C (PLC)-IP_3_ signaling pathway is well-known in the terms of calcium mobilization for neutrophils. FMLP, a classics activator of neutrophils, could result in SOCE on neutrophils through this GPCRs-G protein-PLC-IP_3_ signaling pathway [33], [34], [40]. Therefore, to investigate whether the upstream signaling pathway of sulfhydryl modification-induced SOCE was similar to that of FMLP, PMA-activated neutrophils were stimulated by NEM, SNP and FMLP within Ca^2+^-containing medium in different chronological order. Data showed that FMLP (2 µM) had little capacity for inducing NEM (100 µM) or SNP (500 µM)-pretreated [Ca^2+^]_c_ change in PMA-activated human neutrophils (Fig. 5A and B). This inhibitory effect proposed that sulfhydryl modification may induce SOCE through a GPCRs-G protein-PLC signaling pathway similar to that of FMLP. On the contrary, addition of NEM or SNP after FMLP activation still caused a rapid rising of [Ca^2+^]_c_ (Fig. 5C and D). Comparing with results in Figure 5A and B, it indicated that the pathway of GPCRs-G protein-PLC maybe not the only means for sulfhydryl modification-induced calcium mobilization.

**Figure 6:**
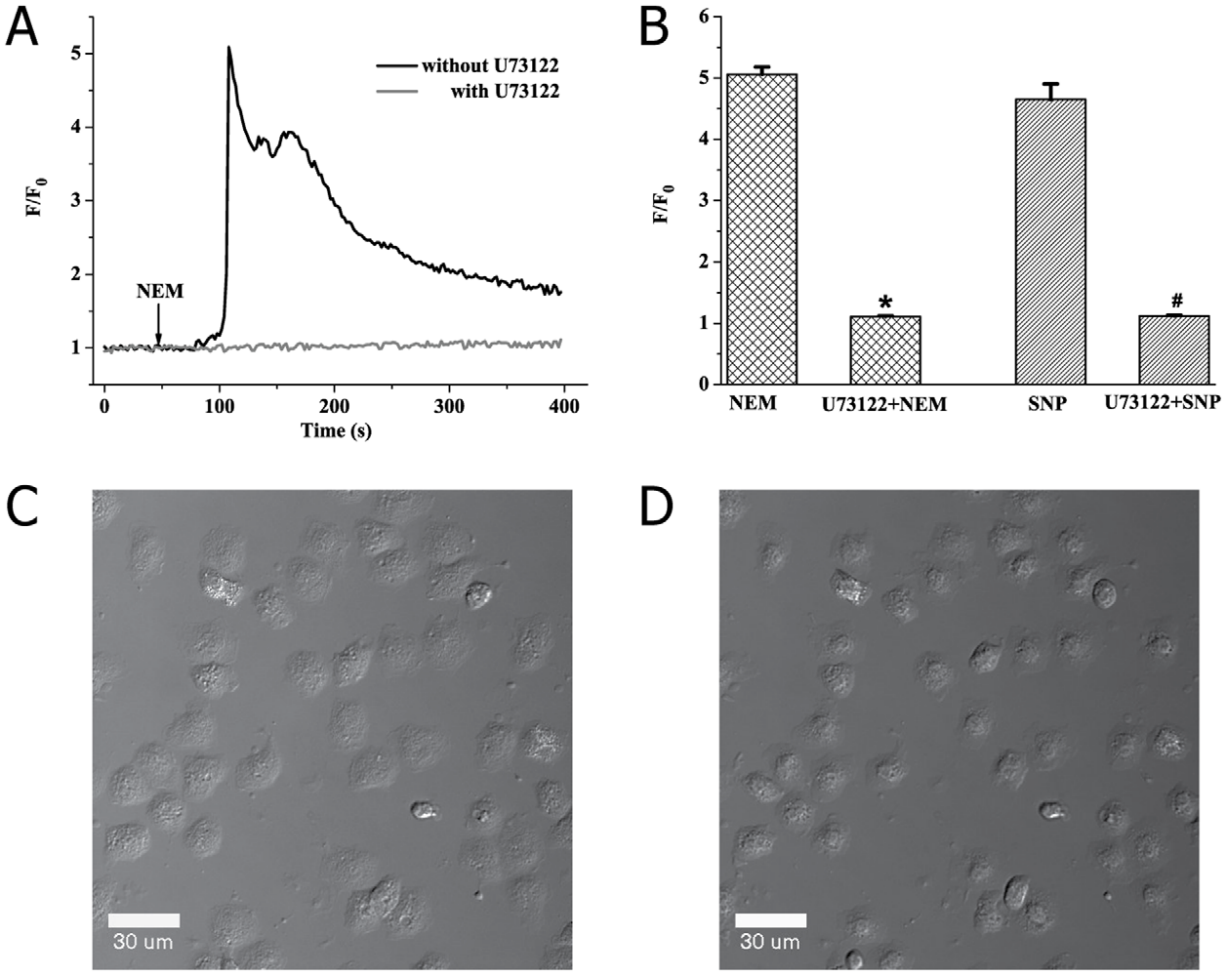
U73122 completely blocks NEM or SNP-induced SOCE and inhibits PMA-elicited spreading of neutrophils. (A) representative tracings of [Ca^2+^]_c_ elevation induced by NEM (100 µM) in the presence or absence of U73122 (10 µM) in Ca^2+^-containing buffer. (B) Statistic results are means ± S.E.M (n = 30 from three independent experiments).^ *^ *P*<0.01, compared with NEM.^ #^ *P*<0.01, compared with SNP. (C) PMA-activated and adherent neutrophils image was obtained by DIC. (D) The effect of U73122 (10 µM) on morphology of PMA-activated neutrophils.

To further study whether sulfhydryl modification-induced SOCE was due to activation of PLC-IP_3_-dependent signaling pathway, PMA-activated neutrophils were stimulated by NEM or SNP in the presence of 1-[6-([(17β)-3-methoxyestra-1,3,5,(10)-trien-17-yl]-amino)-hexyl]- 2,5-dione (U73122), a putative inhibitor of PLC [41], [42]. We found that NEM (100 µM) had no effect on [Ca^2+^]_c_ elevation in Ca^2+^-containing medium when PMA-activated neutrophils were treated with U73122 (10 µM) as summarized in Figure 6A (gray line) and B (maximal value of F/F_0_: 5.062±0.121 for NEM vs. 1.110±0.012 for U73122+NEM, n = 30 cells, P<0.01). Meanwhile, the effect of SNP on [Ca^2+^]_c_ was completely inhibited in Ca^2+^-containing buffer in the presence of U73122 (maximal value of F/F_0_: 4.651±0.253 for SNP vs. 1.121±0.012 for U73122+SNP, n = 30 cells, P<0.01; Fig. 6B). Take together, it was suggested that PLC activation may be involved in the signaling transduction mechanism of NEM or SNP-induced SOCE. Besides, we clearly observed that U73122 resulted in morphological contraction of PMA-induced spreading neutrophils (Fig. 6C and D).

## Discussion

There are a variety of agonists can activate resting neutrophils *in vitro*, including PMA, FMLP, leukotriene B_4_, interleukin-8 and platelet-activating factor [1], [43], [44]. PMA, a diacylglycerol (DAG) analog, specifically activates PKC which in turn activates NADPH oxidase, resulting in stimulation of the respiratory burst of neutrophils [29], [30]. Although PMA is not a physiological neutrophil activator, it was widely used as a model stimulus to study inflammatory responses of neutrophils in vitro experiments [45]–[48]. There are two main reasons for us to select PMA as an activator of neutrophils. First, PMA activates neutrophils without an apparent rising of [Ca^2+^]_c_ [29], [49], [50] and maintains stability of [Ca^2+^]_c_ basal level before stimulation, as shown in all our experiments of [Ca^2+^]_c_ measurement. Second, the activation of the human neutrophils by PMA leads to depolarization of the plasma membrane [29], [51] as seen in Figure 1B, as well as inhibiting spontaneous polarization-induced random migration. Both these attributes ease measurement of [Ca^2+^]_c_ in single cell level with fluorescence microscopy. On the contrary, FMLP, a physiological neutrophil activator, not only caused strong calcium mobilization but also induced obvious migration of neutrophils, thereby complicating measurements of [Ca^2+^]_c_ change in single cell level.

Our studies using TG and FMLP firstly demonstrated that the mechanism of SOCE still exist in PMA-activated neutrophils (Fig. 1). TG, a specific inhibitor of sarcoplasmic/endoplasmic reticulum Ca^2+^ ATPases [32], causes calcium stores depletion and calcium entry via SOCE pathway in many types of cells including neutrophils [31]. FMLP, a chemotactic tripeptide, is well known as a potent and classic SOCE activator in neutrophils [33], [34]. We subsequently found that NEM or SNP induced significant rising of [Ca^2+^]_c_ in PMA-activated neutrophils no matter whether calcium was in the buffer or not (Fig. 2A, 2B, 2C). SNP had no effect on [Ca^2+^]_c_ when PMA-activated neutrophils were pretreated with NEM (Fig. 2B). Readdition of external Ca^2+^ led to a further calcium entry event after activation of NEM or SNP (Fig. 2D and E), indicating calcium entry should be store-depletion-dependent. Besides, we found that addition of 1 mM external calcium did not induce [Ca^2+^]_c_ increase in Ca^2+^-free PMA-activated neutrophils without any stimulation, and subsequently adding NEM (100 µM) to achieve an apparent SOCE observed (Fig. 2F). Moreover, we found that NEM or SNP did not induced change of [Ca^2+^]_c_ in resting human neutrophils which were performed in suspension as shown in Figure 3. In conclusion, these results indicated that NME and SNP could induce a believable response for SOCE via sulfhydryl modification in PMA-induced activation-dependent human neutrophils.

2-APB, a specific inhibitor of IP_3_ receptor [35]–[38], abolished NEM-induced calcium release in a concentration-dependent manner (Fig. 4A and B). 2-APB also had the same effect on SNP-induced [Ca^2+^]_c_ elevation in PMA-activated neutrophils (data not shown). Besides, much research has demonstrated that 2-APB was a reliable blocker of SOCE in recent years [9], [38], [39]. Gill *et al* reported that 2-APB showed obvious inhibitory effect on SOCE at 75 µM [52]. In our experiments, application of 2-APB (75 µM) completely inhibited TG, NEM and SNP-induced SOCE in PMA-activated human neutrophils (Fig. 1E, F and Fig. 4). Moreover, we found that ruthenium red, a specific inhibitor of ryanodine receptor-sensitive stores [53], had no effect on NEM or SNP-induced calcium release PMA-activated neutrophils (data not shown). Considered together, these data further indicated that NEM and SNP resulted in calcium entry through IP_3_ receptor-sensitive stores-operated pathway in PMA-activated human neutrophils.

In addition, it is easy to find that we use a relatively high an order (2 µM) of magnitude higher than usual-PMA-dose to activate neutrophils from the resting state. It should be important to see whether the same phenomenon occurs at a submicromolar concentration. First, we found that 200 nM PMA (30 min at 37°C) could result in neutrophils obvious spreading on glass. Under this condition, NEM (100 µM) or SNP (500 µM) caused obvious [Ca^2+^]_c_ increase (data not shown). However, further result showed that neutrophils could not spread on glass evidently when neutrophils were preincubated with 200 nM for 5 min at 37°C. Subsequent application of NEM or SNP had no obvious effect on [Ca^2+^]_c_ (data not shown). It could be proposed that the activation ability of PMA on neutrophils were concentration and activation time-dependent. Lower concentration of PMA used, more activation time needed. The degree of neutrophils adhesion on glass substrate may have effect on sulfhydryl modification-induced [Ca^2+^]_c_ change.

Having known this, we sought to offer an explanation as to the upstream signaling pathway of NEM and SNP induced-SOCE. For this aim, we firstly studied the effect of FMLP on NEM or SNP-induced SOCE in PMA-activated neutrophils. It is well known [33], [34], [42] that FMLP, a classic activator of neutrophils, can cause stimulation of GPCRs on neutrophils, with consequent activation of PLC and generation of IP_3_ and DAG by breakdown of membrane phosphatidyl inositol 4,5-bisphosphate. Finally, IP_3_ interacts with Ca^2+^-mobilizing receptors, resulting in IP_3_ receptor-sensitive calcium stores release and SOCE. Our data showed that NEM or SNP blocked calcium signaling induced by subsequent stimulation of FMLP (Fig. 5A and B). In contrast, FMLP failed to inhibit calcium elevation caused by subsequent activation by NEM or SNP in PMA-activated neutrophils (Fig. 5C and D). Together, these data proposed that NEM or SNP-induced calcium mobilization in PMA-activated neutrophils may involve the same signaling pathway as that of FMLP, namely the GPCRs-G protein-PLC-IP_3_ pathway. However, this may not be the only pathway for NEM or SNP-induced SOCE.

Furthermore, it was found that demonstrated that U73122 completely blocked NEM or SNP-induced calcium mobilization in Ca^2+^-containing medium (Fig. 6A and B). U73122 is a putative and specific PLC inhibitor which has become an important tool in establishing the link between PLC activation and intracellular calcium signaling [40], [41]. However, we must admit that the inhibitory mechanism of U73122 on NEM or SNP-induced SOCE seems to be problematic because U73122 is a sterol derivative of NEM. Therefore, such data just indicated possible roles for PLC in NEM or SNP-induced calcium responses. In addition, we directly observe an interesting phenomenon that U73122 caused morphological contraction of PMA-induced spreading of neutrophils in glass substrate observed by DIC microscopy (Fig. 6C and D), which is in accordance with observations by Smith *et al* who reported that U73122 could cause a inhibition of PMA-triggered neutrophils adhesion on fibronectin and fetal bovine serum-coated plates [54]. This result also suggested that sulfhydryl modification-induced [Ca^2+^]_c_ elevation may associate with the degree of neutrophils adhesion on glass substrate similar to that of PMA.

Interestingly, Wang *et al* showed that NO donor GEA3162 and NEM could induce calcium entry through a non-store-operated mechanism via direct sulfhydryl modification in resting rat neutrophils which were performed in suspension [22]–[24]. Gill *et al* also reported that NO donors and NEM caused non-store-operated calcium entry through sulfhydryl modification in DDT_1_MF-2 cells [25]–[27]. It is obvious that activation of neutrophils by PMA adherent to a glass substrate give a different result of sulfhydryl modification-induced calcium mobilization compared to resting neutrophils. Other studies reported that neutrophils would give different physiological or psychological responses to stimuli depending on their being in the activation or resting state [44], [55]. We analysed that activation of neutrophil respiratory burst may be implicated in the different regulation of [Ca^2+^]_c_ induced by sulfhydryl modification. To address this hypothesis, diphenylene iodonium (DPI), a putative inhibitor of the respiratory burst-generating NADPH oxidase, was used in the present work. The results provided evidence that DPI had a inhibitory effect on NEM-induced SOCE in PMA-treated neutrophils (data not shown). Our previous report also demonstrated that DPI could block SNP-induced calcium release via S-Nitrosylation [28]. Meanwhile, U73122 completely inhibited NEM and SNP-induced SOCE in PMA-activated neutrophils (Fig. 6A and B). It was reported that U73122 could inhibit PMA-elicited adhesion-dependent respiratory burst [54]. Moreover, We also found that NEM and SNP did not induce obvious [Ca^2+^]_c_ increase when neutrophils was preincubated with PMA just at submicromolar concentration for little time at 37°C (data not shown). These evidence suggested the activation degree of respiratory burst may contribute to generation of sulfhydryl modification-induced SOCE in neutrophils. However, further study is still needed to elucidate the relationship between activation of respiratory burst-generating NADPH oxidase and sulfhydryl modification.

In summary, we clearly demonstrated that NEM, a thiol-alkylating agent, and SNP, a donor of exogenous NO, induced obvious calcium entry through IP_3_-sensitive store-operated pathway via sulfhydryl modification in PMA-induced activation-dependent human neutrophils. Since SOCE plays a crucial role in neutrophils, this study on investigating sulfhydryl modification-induced SOCE may help contribute towards understanding mechanisms of [Ca^2+^]_c_ regulation surrounding neutrophil inflammatory reactions.

## Materials and Methods

### Ethics Statement

The study was reviewed and approved by the Ethics Committee of Nankai University. All participants gave written informed consent.

### Reagents

SNP, NEM, PMA, FMLP, 2-APB, TG, U73122, DPI, EGTA, Histopaque 1077 and 1119 solutions, Hoechst 33342, trypan blue were obtained from Sigma–Aldrich (St. Louis, MO, USA). Fluo-3 acetoxymethyl ester (Fluo-3 AM) was from Gibco (Gaithersburg, MD, USA).

### Preparation of human neutrophils

Neutrophils were obtained from the blood of normal healthy adults by step-density gradient centrifugation over Histopaque 1077 and 1119 solutions at 500×g for 15 min. The cells were >96% viable as assessed by trypan blue exclusion and had a purity of >95% as demonstrated by Hoechst 33342 nuclear staining. Cells were suspended in HBSS (145 mM NaCl, 5 mM KCl, 1 mM CaCl_2_, 1 mM MgCl_2_, 10 mM Hepes and 10 mM glucose, PH adjusted to 7.4 with NaOH 1 mM) and then kept in an ice bath before use.

### Microscopy

For all imaging experiments, cells in chamber (volume, 1 mL) were observed microscopically at 37°C by using a temperature-controlled stage. Fluorescence was performed with a fluorescent microscope (Axio observer D1, Carl Zeiss, Germany) with a 100 W mercury lamp. A shutter (VS25S2ZM1, Uniblitz, USA) was equipped behind the mercury lamp to control the exposure time and the interval time for fluorescence imaging. Fluorescence signaling was detected by an electron multiplying charge coupled device (EMCCD) (DU-897D-CS0-BV, Andor, U.K., ≈−80°C) which was connected to the left exit side of microscope. The EMCCD was controlled by MetaMorph software version 7.1 (Universal Imaging Corp., USA). DIC and fluorescence images were collected by this microscopy system.

### Measurement of [Ca^2+^]_c_

Human neutrophils were incubated with calcium-sensitive probe Fluo-3 AM 4 µM for 30 min at 37°C in HBSS. For single cell calcium imaging, [Ca^2+^]_c_ was measured by the micro-imaging system as shown above. Fluo-3 AM-loaded cells were excited by a mercury lamp with a 485/20 nm excitation filter, and fluorescence was collected by a fluar oil objective with a 510 nm long-pass dichroic mirror and a 540/50 emission filter. Each fluorescence image was acquired for 50 msec with a 2 s interval between frames. The obtained images were quantitatively analyzed for changes of fluorescence intensities within region of interest. [Ca^2+^]_c_ change was represented by relative fluorescence intensity (F/F_0_, intensity after stimulation/basal intensity before stimulation). At least 30 individual cells were selected from three independent experiments, with one characteristic [Ca^2+^]_c_ trace plotted to represent >10 similar traces. Besides, Ca^2+^-free HBSS was prepared by substituting MgCl_2_ for CaCl_2_ at the same concentration, with 2 mM EGTA. For [Ca^2+^]_c_ measurement in suspension, Fluo-3 AM-loaded neutrophils were suspended in HBSS to 1×10^6^cells/mL. Fluorescence was monitored with a spectrofluorometer (FSL920, Edinburgh, U.K.) at 530/30 nm with excitation at 488 nm.

### Statistical analysis

All data were presented as means ± standard error of the mean (S.E.M.). The statistical comparison between groups was carried out using Student’s *t*-test. *P*<0.05 was considered statistically significant.
